# Handling Uncertainty in Cost-Effectiveness Analysis: Budget Impact and Risk Aversion

**DOI:** 10.3390/healthcare9111419

**Published:** 2021-10-22

**Authors:** Pedram Sendi, Klazien Matter-Walstra, Matthias Schwenkglenks

**Affiliations:** 1 Institute for Clinical Epidemiology, Basel University Hospital, Spitalstrasse 12, 4031 Basel, Switzerland; 2 Institute of Pharmaceutical Medicine (ECPM), University of Basel, Klingelbergstrasse 61, 4056 Basel, Switzerland; klazien.matter@hispeed.ch (K.M.-W.); m.schwenkglenks@unibas.ch (M.S.)

**Keywords:** economic evaluation, cost-effectiveness analysis, risk-aversion, budget impact, uncertainty, health care costs, health outcomes

## Abstract

Methods to handle uncertainty in economic evaluation have gained much attention in the literature, and the cost-effectiveness acceptability curve (CEAC) is the most widely used method to summarise and present uncertainty associated with program costs and effects in cost-effectiveness analysis. Some researchers have emphasised the limitations of the CEAC for informing decision and policy makers, as the CEAC is insensitive to radial shifts of the joint distribution of incremental costs and effects in the North-East and South-West quadrants of the cost-effective plane (CEP). Furthermore, it has been pointed out that the CEAC does not incorporate risk-aversion in valuing uncertain costs and effects. In the present article, we show that the cost-effectiveness affordability curve (CEAFC) captures both dimensions of the joint distribution of incremental costs and effects on the CEP and is, therefore, sensitive to radial shifts of the joint distribution on the CEP. Furthermore, the CEAFC also informs about the budget impact of a new intervention, as it can be used to estimate the joint probability that an intervention is both affordable and cost-effective. Moreover, we show that the cost-effectiveness risk-aversion curve (CERAC) allows the analyst to incorporate different levels of risk-aversion into the analysis and can, therefore, be used to inform decision-makers who are risk-averse. We use data from a published cost-effectiveness model of palbociclib in addition to letrozole versus letrozole alone for the treatment of oestrogen-receptor positive, HER-2 negative, advanced breast cancer to demonstrate the differences between CEAC, CEAFC and CERAC, and show how these can jointly be used to inform decision and policy makers.

## 1. Introduction

Difficulties in estimating a confidence interval for the incremental cost-effectiveness ratio have led to the development of two related approaches to handle uncertainty in cost-effectiveness analysis, namely the net benefit approach and the cost-effectiveness acceptability curve (CEAC) [1,2]. The net monetary benefit (NMB) approach linearly transforms the results of a cost-effectiveness analysis by multiplying the incremental effects of an intervention with the ceiling ratio, often interpreted as the maximum willingness to pay per health outcome, and subtracting the costs thereof [2,3,4]. The analyst can then estimate a confidence interval for the expected NMB without encountering the technical difficulties associated with estimating a confidence interval for a ratio statistic [4]. However, the most widely used method to analyse and present uncertainty in cost-effectiveness analysis is the CEAC [1,5,6]. When constructing the CEAC, the ceiling ratio, representing a line through the origin on the cost-effectiveness plane (CEP), is rotated anticlockwise from zero to infinity and the proportion of the joint distribution of incremental costs and effects lying to the South of the ceiling ratio is estimated as the probability that the new intervention is cost-effective [6]. The CEAC has been introduced almost three decades ago and has become standard repertoire for analysing and presenting uncertainty in trial-based as well as model-based cost-effectiveness analyses.

However, some authors have pointed out that the CEAC is insensitive to radial shifts of the joint distribution of incremental costs and effects in the North-East and South-West quadrants of the CEP [7]. These distributions would vary in terms of incremental costs and effects but would have the same correlation between costs and effects and the same coefficient of variation (i.e., ratio of standard deviation to the mean) [8]. As noted by Fenwick and Briggs, however, insensitivity to radial shifts on the CEP is not a limitation of the CEAC per se, but implied in estimating the ratio of incremental costs to effects, as information about the size of the program is lost [9]. Another, less often used tool for analysing the joint distribution of incremental costs and effects on the CEP, namely the cost-effectiveness affordability curve (CEAFC), does indeed capture radial shifts of the joint distribution on the CEP and, therefore, addresses the limitation of the CEAC mentioned above [8]. In addition to the ceiling ratio, the CEAFC makes use of a budget constraint reflected as a horizontal line on the CEP and, therefore, captures both dimensions of the joint distribution on the CEP.

Another limitation of the CEAC, as discussed by Koerkamp et al. [7], may be that it is not very helpful to inform decision-makers who are risk-averse. Risk-neutral decision makers would base their decision on expected costs and effects alone, hence making methods to handle and present uncertainty in cost-effectiveness analysis irrelevant [6]. However, decision makers may hold limited budgets and are hence incentivised to minimising the risk of exceeding the available budget, or they may need to meet health outcome targets and, hence, may want to minimise the risk of underperformance in health outcomes [10,11,12]. Different approaches have been suggested to include the risk posture of decision-makers in cost-effectiveness analysis by incorporating a preference function, such as a utility function into the analysis [13,14,15]. However, these approaches require that the decision-maker is explicit about his preference function, which is rarely the case in practice [11]. It might therefore be helpful to analyse uncertain costs and effects in cost-effectiveness analysis in a way that incorporates risk-aversion but does not require an explicit preference function to be derived from the decision-maker. The recently introduced cost-effectiveness risk-aversion curve (CERAC) may help to achieve this goal [16].

In the present article we, therefore, demonstrate the application of the CEAC, CEAFC and CERAC using a hypothetical example, and a real-world example based on a published Markov model evaluating the cost-effectiveness of palbociclib in addition to letrozole versus letrozole alone for the treatment of oestrogen-receptor positive, HER-2 negative, advanced breast cancer [17].

## 2. A Hypothetical Example

**Figure 1:**
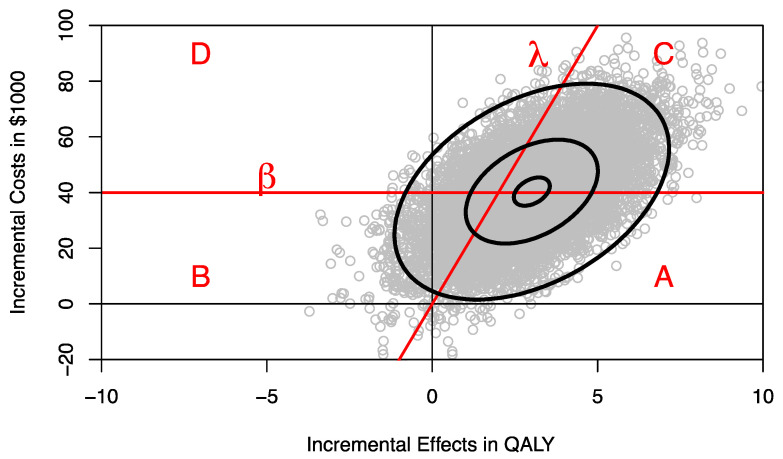
Incremental costs and effects of program F versus program E on the cost-effectiveness plane. *λ* denotes the ceiling ratio, *β* denotes the budget constraint. A denotes the area where the intervention is both affordable and cost-effective, B denotes the area where the intervention is affordable but not cost-effective, C denotes the area where the intervention is cost-effective but not affordable, D denotes the area where the intervention is neither affordable nor cost-effective.

In this section we use a hypothetical example to technically demonstrate the concept of CEAFC and CERAC. Consider two health care programs F and E with mean per-patient costs and effects of $90,000 and 13 quality-adjusted life-years (QALYs) and $50,000 and 10 QALYs, respectively, as shown in Table 1. The standard deviations for costs and effects and the correlation between costs and effects for each program are also shown in Table 1. The joint distribution of incremental costs and effects is depicted in Figure 1 and was estimated by sampling 10,000 times from the respective distributions.

The joint distribution of incremental costs and effects on the cost-effectiveness plane (CEP) can be separated into four areas divided by the ceiling ratio λ, representing the decision-maker’s maximum willingness to pay (WTP) per QALY, and the budget constraint line β, defined by a horizontal line on the CEP (Figure 1) [8]. The proportion of the joint distribution below the ceiling ratio λ represents the probability that program F is cost-effective compared to program E, and the proportion of the joint distribution below the budget constraint β represents the probability that introducing program F to replace program E is within the budget constraint and hence affordable. The ceiling ratio λ and the budget line β divide the joint distribution of incremental costs and effects into four parts as shown in Figure 1:

(i) Area A where the program is both affordable and cost-effective;
(ii) Area B where the program is affordable but cost-ineffective;
(iii) Area C where the program is not affordable but cost-effective;
(iv) Area D where the new program is neither affordable nor cost-effective.

**Figure 2:**
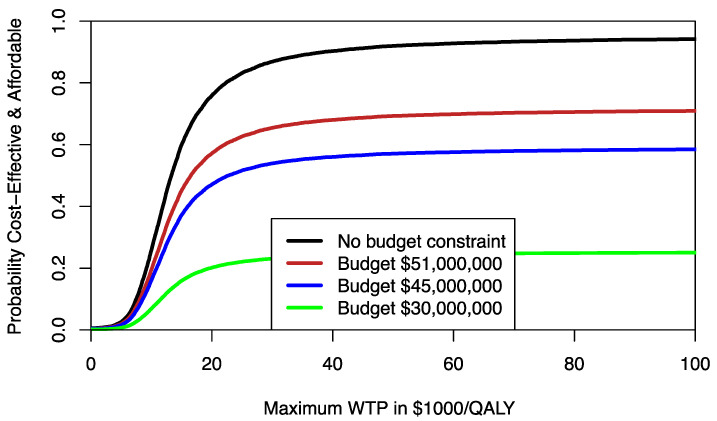
Cost-effectiveness affordability curves (CEAFCs) for different budget constraints comparing program F to program E. Without any budget constraint, the CEAFC corresponds to the CEAC.

Decision makers are likely to be most interested in area A [8]. It may be helpful to estimate area A for different budget constraints. For any given budget constraint β, we can rotate the ceiling ratio anticlockwise and estimate the probability that program F compared to program E is both cost-effective and affordable, resulting in a CEAFC [8]. For example, assuming that 1000 patients would need the treatment provided by program F, we can use different percentile levels of the incremental cost distribution to estimate the respective CEAFC. In Figure 2, the CEAFC is estimated for 1000 patients and a budget constraint of $30 million (25% percentile of incremental costs), $45 million (median of incremental costs), and $51 million (75% percentile of incremental costs). Another approach would be to define an a priori budget and estimate the respective CEAFC. The CEAFC not only informs about the budget impact and return on investment in a healthcare program, but also captures any shifts of the joint distribution in the North-East quadrant of the CEP [7,8]. For a more detailed discussion of the CEAFC we refer to Sendi and Briggs [8].

Decision-makers, however, may not only be concerned with the affordability and cost-effectiveness of a healthcare program, but may also exhibit different levels of risk-aversion [16]. It may, therefore, be helpful to also calculate risk-adjusted performance measures that include risk-aversion when analysing cost-effectiveness models [16,18]. Although a number of methods have been suggested to take risk-aversion into account, most of these rely on an explicit preference function, which may be difficult to elicit in practice [13,14,15]. A recently proposed method, the CERAC, may help to inform decision makers with risk-aversion without the need to explicitly derive a preference function [16]. The CERAC estimates the net benefit to risk ratio of a program for a large number of ceiling ratios [16]. The net benefit to risk ratio *S_NMB_* as previously defined can be written as

(1) $$S_{NMB}=\frac{\mu_{NMB}}{DD_{NMB}}\;$$

where

(2) $$\mu_{NMB}=\mu_{E}\;\cdot \lambda -\mu_{C}\;$$

where *μ_NMB_* denotes the expected NMB of a program, *μ_E_* denotes mean effect, *μ_C_* mean cost of a program, and λ the ceiling ratio. *DD_NMB_* denotes the downside deviation, defined as

(3) $$DD_{NMB}=\sqrt{\frac{1}{n}\overset{n}{\underset{i=1}{\sum }}{\left(NMB_{i}-\mu_{NMB}\right)}^{2}f\left(t\right)\;}$$

$$\begin{array}{c} f\left(t\right)=1\;if\;NMB_{i}<\mu_{NMB}\\ f\left(t\right)=0\;if\;NMB_{i}\geq \mu_{NMB} \end{array}$$

where *NMB_i_* denotes a sample observation, which may be derived, for example, from bootstrapping mean costs and effects of a program [16]. The root-mean-square of all sample observations corresponds to the *DD_NMB_*. The *S_NMB_* Equation (1) penalises the expected NMB of a program *μ_NMB_* for its “bad” risk (i.e., its downside deviation *DD_NMB_*). Recalling Equation (3), *DD_NMB_* will be higher either if the number of observations *n* below *μ_NMB_* is higher and/or if the magnitude of deviations below *μ_NMB_* is higher. The method allows to include different levels of risk-aversion by defining a different minimally acceptable NMB for the downside deviation. For example, instead of penalising expected NMB for the downside deviation relative to the mean, a less risk-averse decision-maker may decide that any NMB below the 25% percentile of the NMB distribution denoted as $\eta_{25_{NMB}}$ would be considered as underperformance, and we would rewrite Equation (3) as

(4) $$DD_{NMB}=\sqrt{\frac{1}{n}\overset{n}{\underset{i=1}{\sum }}{\left(NMB_{i}-\eta_{25_{NMB}}\right)}^{2}f\left(t\right)\;}$$

$$\begin{array}{c} f\left(t\right)=1\;\mathrm{if}\;NMB_{i}<\eta_{25_{NMB}}\\ f\left(t\right)=0\;\mathrm{if}\;NMB_{i}\geq \eta_{25_{NMB}} \end{array}$$

Alternatively, when comparing different health care programs, the decision maker may want to define a common minimally acceptable NMB across all programs to estimate the *DD_NMB_* relative to a common yardstick. The concept of downside deviation is very versatile and powerful, and allows the decision-maker to define a constant or varying threshold level for a minimally acceptable NMB below which an intervention would be considered as providing insufficient economic value. For example, if there are three treatment options for the treatment of lung cancer, the analyst may want to define the 25% percentile of the NMB distribution for surgery/chemotherapy (intervention 1) as the minimally acceptable NMB, and use that same threshold level to also estimate *DD_NMB_* for radiotherapy/chemotherapy (intervention 2), and radiotherapy/chemotherapy/immunotherapy (intervention 3).

**Figure 3:**
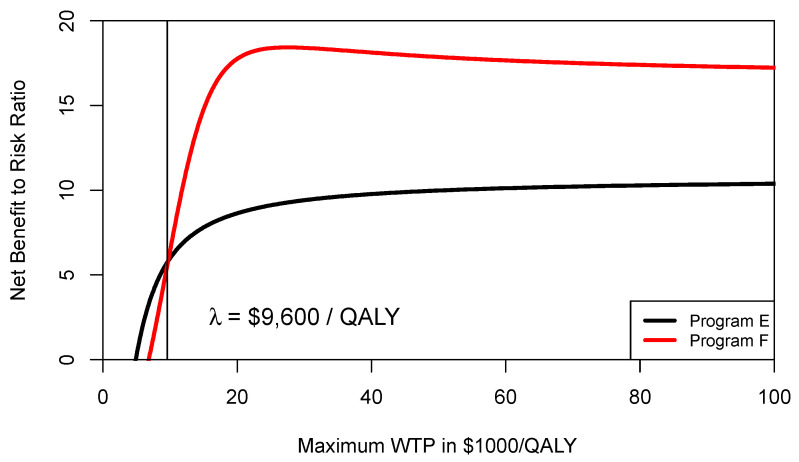
Cost-effectiveness risk-aversion curve (CERAC). At a ceiling ratio of $9600 per QALY, program F becomes preferable to program E (Table 1) as it offers more expected return per unit of downside risk.

However, in this section and for demonstration purposes, we use Equation (3) to estimate the CERAC for program F and program E, which rather implies a higher level of risk-aversion [16,19]. We construct the CERAC by calculating the net benefit-to-risk ratio *S_NMB_* for each individual program and for all possible ceiling ratios λ by sampling 10,000 times from the distributions defined in Table 1. Figure 3 shows the CERAC for program F and program E. As can be seen from Figure 3, at a ceiling ratio of $9600 per QALY, program F becomes the preferred strategy and offers a higher net benefit to risk ratio. In other words, the threshold level where program F becomes preferable is different when comparing the CEAC ($13,333/QALY) with the CERAC ($9600/QALY).

## 3. The Example of Breast Cancer Treatment

In the section above we used a simple hypothetical example to illustrate the concept of CEAFC and CERAC. In this section we use results from a validated and peer-reviewed published model on the cost-effectiveness of a combination of palbociclib in addition to letrozole compared to letrozole alone for the treatment of oestrogen-receptor positive, HER2 negative, advanced breast cancer to estimate the CEAC, CEAFC and CERAC [17].

The PALOMA-1 phase II trial showed that in patients without prior systemic treatment for metastatic breast cancer, a combination of palbociclib and letrozole (PALLET) compared to letrozole (LET) alone increased progression-free survival from 10.2 months to 20.2 months, nearly a two-fold increase [17]. The results of the PALOMA-1 trial were used to estimate the cost-effectiveness of PALLET compared to LET from a Swiss healthcare perspective using a Markov model [17]. A lifelong time horizon was adopted, and effects expressed in QALYs and costs in 2015 Swiss Francs (CHF) [17]. The joint impact of uncertain model input parameters on lifetime costs and effects were evaluated using a probabilistic sensitivity analysis (PSA) based on 10,000 samples in a second-order Mote Carlo simulation [17]. In the base case analysis, mean cost and effects for PALLET were of CHF 501,105 ($US 537,447) and 3.33 QALYs; for LET mean cost and effects were CHF 158,665 ($US 170,489) and 2.19 QLAYs. PALLET compared to LET, therefore, led to an increase in 1.14 QALYs at an additional cost of CHF 342,440 ($US 367,959), resulting in a cost-effectiveness ratio of CHF 301,227 ($US 323,674) per QALY gained [17].

**Figure 4:**
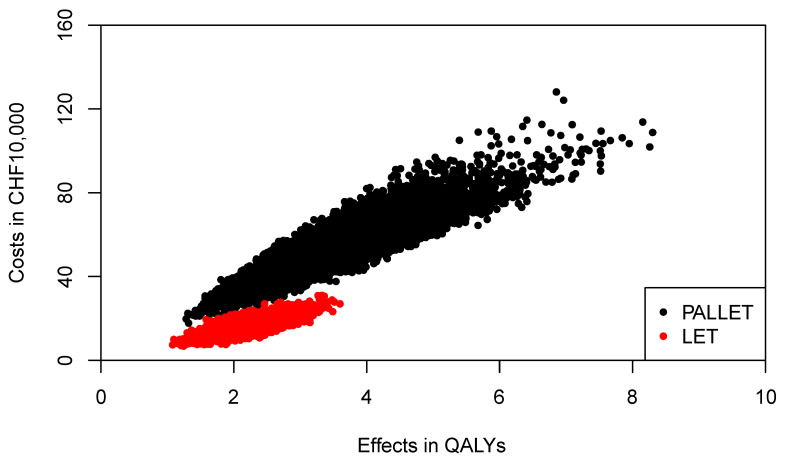
Joint distribution of total costs and effects of PALLET (palbociclib and letrozole) versus LET (letrozole) in patients with metastatic ER + HER2- breast cancer. One Swiss Franc (CHF) corresponds to $US 1.07.

**Figure 5:**
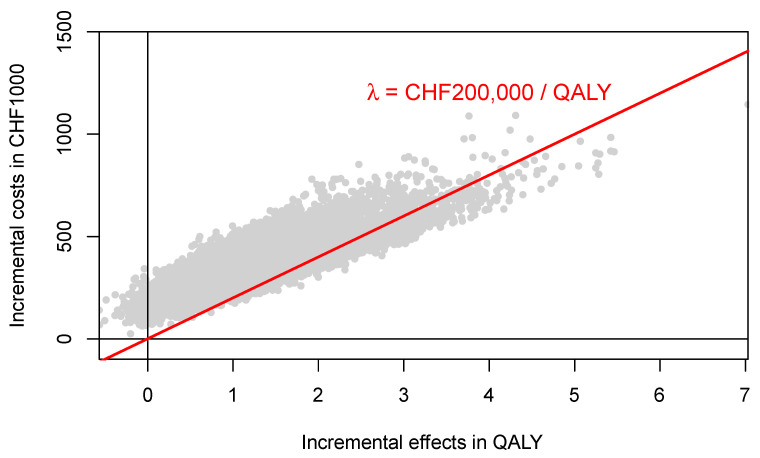
Incremental costs and effects of PALLET (palbociclib and letrozole) versus LET (letrozole) in patients with metastatic ER + HER2- breast cancer. The red line represents the ceiling ratio. The proportion of samples below the ceiling ratio represents the probability that PALLET is cost-effective at CHF 200,000 per QALY, which is 11%. One CHF (Swiss Franc) corresponds to $US 1.07.

**Figure 6:**
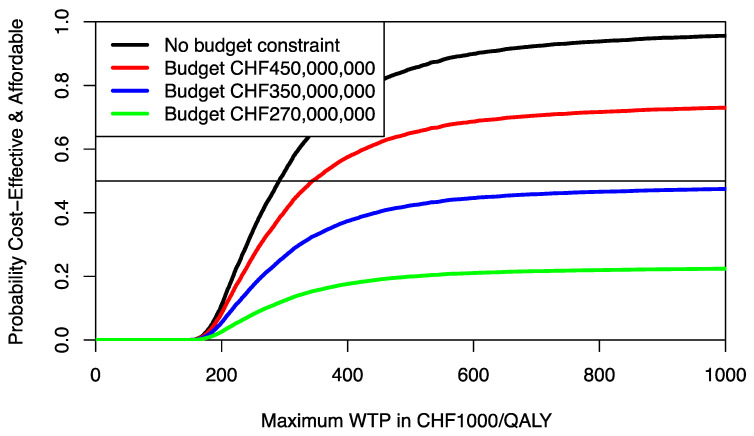
Cost-effectiveness affordability curves (CEAFC) of PALLET (palbociclib and letrozole) versus LET (letrozole) in patients with metastatic ER + HER2- breast cancer. In the absence of a budget constraint, the CEAFC corresponds to the CEAC. The horizontal line at probability 0.5 represents the anticipated minimal joint probability an intervention is both cost-effective and affordable.

The joint distributions of costs and effects of PALLET and LET are shown in Figure 4. As can be seen, PALLET has a much higher variability in costs and effects than LET. The distributions for costs and effects for PALLET are highly skewed to the right, and the Shapiro-Wilk Test for normality is *p* < 0.0001 for the costs and effects for both PALLET and LET, indicating that all distributions are not normal. Hence, the respective bivariate distributions are also not normal. The joint distribution of incremental costs and effects of PALLET versus LET is shown in Figure 5. As can be seen, at a ceiling ratio of CHF 200,000 ($US 214,904) per QALY, the probability that the PALLET is cost-effective is only 11%. The CEAC (corresponding to the CEAFC without any budget constraint) is shown in Figure 6. The budget impact of PALLET is substantial. Assuming a cohort of 1000 patients in whom PALLET would be prescribed, which roughly corresponds to the number of deaths from ER positive and HER2 negative metastatic breast cancer over two years in Switzerland [17], the required additional budget would be approximately CHF 342,000,000 ($US 376,486,000). As can be seen in Figure 6, assuming an available budget of CHF 450,000,000, ($US 483,534,000) corresponding to the 75% percentile of the incremental cost distribution (red CEAFC in Figure 6), the decision-maker’s maximum WTP per QALY must be at least CHF 350,000 ($US 376,082) per QALY, in order for PALLET to be both affordable and cost-effective with a joint probability greater than 50%. With an available budget of CHF 270,000,000 ($US 290,120,00), corresponding to the 25% percentile of the incremental cost distribution (green CEAFC in Figure 6), the joint probability that the intervention is both affordable and cost-effective is always low and barely exceeds 20%, even if the decision-maker were willing to pay CHF 1,000,000 ($US 1,074,520) per QALY gained.

**Figure 7:**
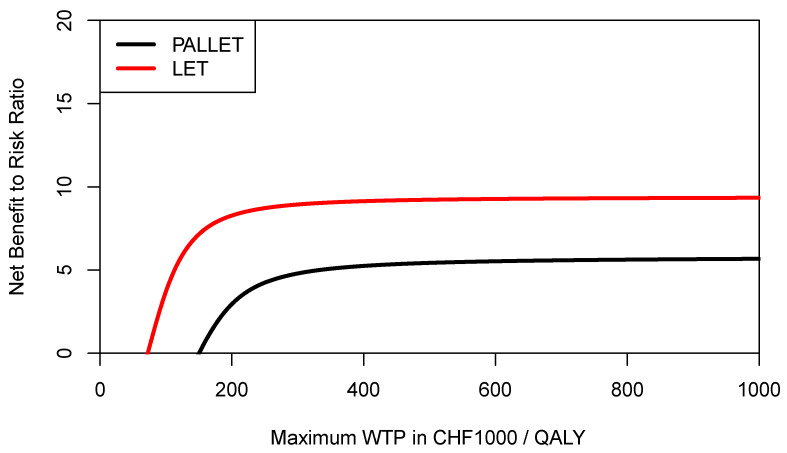
Cost-effectiveness risk-aversion curve (CERAC) of PALLET (palbociclib and letrozole) versus LET (letrozole) in patients with metastatic ER + HER2- breast cancer. CERACs are estimated using Equation (3) as downside deviation.

In order to inform a risk-averse decision-maker, we may also want to construct the respective CERACs. Using Equation (3) to estimate downside deviation for PALLET and LET, the CERACs are constructed by calculating the net benefit to risk ratio for each value of the ceiling ratio as shown in Figure 7. As can be seen, LET always has a higher net benefit to risk ratio than PALLET and would, therefore, be preferred by a risk-averse decision-maker.

**Figure 8:**
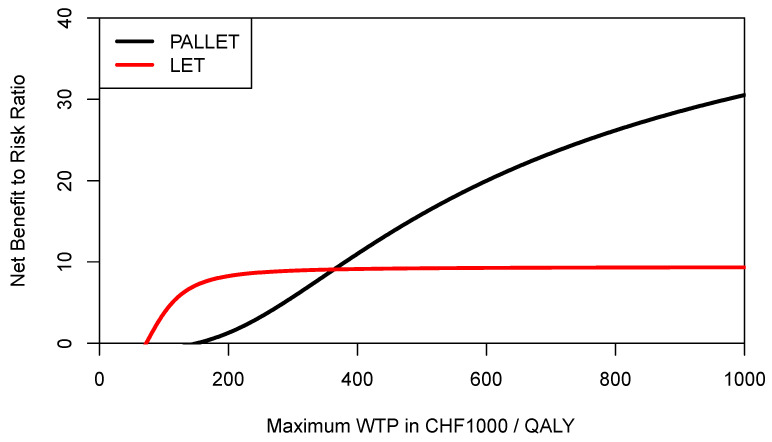
Cost-effectiveness risk-aversion curve (CERAC) of PALLET (palbociclib and letrozole) versus LET (letrozole) in patients with metastatic ER + HER2- breast cancer. CERACs are estimated using the mean NMB of LET to estimate the downside deviation for both PALLET and LET.

**Figure 9:**
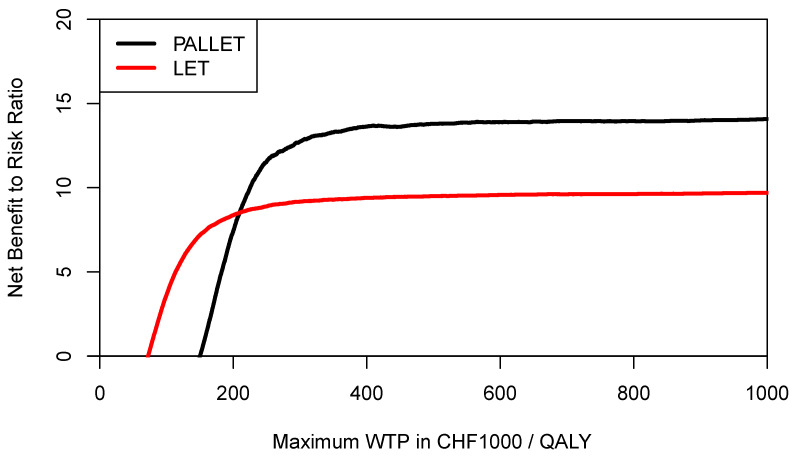
Cost-effectiveness risk-aversion curve (CERAC) of PALLET (palbociclib and letrozole) versus LET (letrozole) in patients with metastatic ER + HER2- breast cancer. CERACs are estimated using the 25% percentile of the NMB distribution for PALLET and the median of the NMB distribution for LET to estimate downside deviation. The two CERACs cross at CHF 209,600 ($US 225,219) per QALY where PALLET becomes preferable.

However, recalling Equations (1) and (3), the expected NMB of each program is penalised for its downside deviation *DD_NMB_* relative to the mean NMB, which is of course different for each program for a given ceiling ratio. As also shown in Equation (4) for the example of the 25% percentile of the NMB distribution, the definition of downside deviation *DD_NMB_* offers much more flexibility, and a decision maker may want to use a common yardstick for both programs below which any NMB would be considered as providing insufficient economic value (i.e., underperformance). Let us assume that for a given ceiling ratio the decision-maker considers any NMB sample observation below the mean NMB of LET as underperformance for both PALLET and LET, then the *DD_NMB_* for PALLET would need to be modified accordingly, and the respective CERACs estimated via simulation are shown in Figure 8. As can be seen, when mean NMB of LET is used as a common yardstick to estimate downside deviation for both programs, then the CERAC for PALLET crosses the CERAC for LET at a ceiling ratio of CHF 363,000 ($US 390,051) per QALY (Figure 8) and becomes the preferred strategy, offering more expected return per unit of downside risk. As another example, let us assume a decision maker rather wants to define any NMB sample observation below the 25% percentile of the NMB distribution of PALLET as underperformance, and at the same time any NMB sample observation below the median of LET as underperformance. These two respective CERACs estimated using simulation are shown in Figure 9. In Figure 9, the CERACs for PALLET and LET cross at CHF 209,600 ($US 225,219) per QALY where PALLET becomes preferable. As shown by these examples, the CERAC is very versatile, and can accommodate a constant or a varying value for the minimally acceptable NMB below which one would consider a program’s return on investment as insufficient. A lower minimally acceptable NMB implicitly reflects a lower degree of risk-aversion.

## 4. Discussion

In the present paper we have shown that the CEAFC and CERAC are helpful tools to inform decision makers about the consequences of funding a new healthcare program [1,8,16]. The CEAFC and CERAC address the limitations of the CEAC pointed out by other authors [7]. The CEAFC not only informs about the budget impact of an intervention, but also captures any radial shifts of the joint distribution of incremental costs and effects in the North-East quadrant of the CEP. Outcomes in the South-West quadrant of the CEP are less common, indicating that resources are released and health outcomes reduced. This rather reflects the policy of removing an existing healthcare program to release resources, which, in turn, can be used to fund new healthcare programs [20,21]. It is certainly noteworthy that a different ceiling ratio may apply in the North-East and South-West quadrant of the CEP [22]. In the North-East quadrant of the CEP, the ceiling ratio represents the decision maker’s maximum WTP per QALY, in the South-East quadrant it represents the decision makers minimum willingness to accept (WTA) to forgo one QALY [22]. Since current evidence suggests that losses are not valued equally as gains, the CEAC may be modified to include a WTP/WTA-disparity, as suggested by Severens et al. [23,24].

The CEAFC has mainly been used in economic evaluations in developing countries with more pressing budget constraints [25,26], but also in dentistry [27] and in a recent evaluation of cancer drugs for HER+ metastatic breast cancer in England [28]. In a review of published studies using the CEAFC, Yi et al. concluded that CEAFCs are underused in developed countries and should be used more often [29]. Information about the size of a program is lost when using cost-effective ratios as the sole criterion for decision making [7,8,9]. A decision-maker may indeed want to maximise the probability that an intervention is both cost-effective and affordable. Ideally, this joint probability should be greater than 50%. It might be argued that it is difficult in developed countries to define an explicit budget constraint. However, the budget constraint used for estimating the CEAFC serves two purposes. First, it is used as a technical instrument to unambiguously locate the joint distribution of incremental costs and effects on the CEP as described above. Second, the decision maker may alternatively want to define the maximally acceptable probability of exceeding the budget constraint, for example, 5% or 10%, similar to accepting a 5% Type I error in hypothesis testing. He can then use this threshold probability level to define *ex post* the anticipated budget needed to fund a health care program.

There may be theoretical objections to using the CEAFC, since with certain costs and effects the incremental cost-effectiveness ratio represents the shadow price of the budget constraint [30]. In other words, when using linear programming to solve a constrained optimisation problem where aggregate health outcomes are maximised, the budget is already a constraint in the optimisation problem [31,32]. However, the constrained optimisation approach assumes that the costs and effects of all programs are known and certain. But the contrary is true in reality. Costs and effects are subject to uncertainty and change, and are not known for all programs funded in a health care system [11]. When costs and effects are uncertain, constrained optimisation does not necessarily lead to a solution where the cut-off point for resource allocation is represented by a cost-effectiveness ratio [11,32]. When, nonetheless, a fixed cost-effectiveness ratio is used as a cut-off point for resource allocation, then this leads to an uncontrolled growth of health expenditures, unrealistically assuming constant marginal opportunity costs [33,34,35].This does not necessarily invalidate the use of a threshold ratio in cost-effectiveness analysis, but its interpretation changes from being the shadow price of the constrained budget to being a measure of return on investment in a health care program [18,36]. Therefore, affordability concerns become even more relevant and further stress the importance of the CEAFC in daily practice to inform decision and policy makers. Of note, an interesting alternative approach to include affordability concerns in cost-effectiveness analysis has been suggested by Lomas, where the ceiling ratio is defined as a function of the program’s budget impact and health opportunity costs [37].

The literature on risk-aversion in cost-effectiveness analysis is quite limited [13,14,15,16,18]. This may be due to the fact that usually a utility function over expected return and risk is assumed, which makes it difficult for general use in practice [11,16]. Decision makers may not easily exhibit their risk-posture, which is indeed needed if a utility function were to be used to describe the trade-off between risk and return. The CERAC has recently been introduced as a means to incorporate risk aversion when analysing uncertain costs and effects in cost-effectiveness analysis without the need of an explicit utility function [16]. The Sortino ratio, a common metric in finance used for measuring risk-adjusted asset performance, has been adapted for its use in health care finance [16]. Investment in a health care program can be interpreted as an investment in a risky asset. The expected return in a health care program, expressed in expected NMB, is then penalised for its downside deviation. The concept of downside deviation is quite versatile, and can be used to define any minimally acceptable NMB as a threshold below which a program’s NMB sample observation would be considered as underperformance [38]. As the examples in this article show, the CERAC allows the decision maker to use the same minimally acceptable NMB for all programs being compared. Or, alternatively, the decision maker can define a different minimally acceptable NMB for each program, hereby expressing differing risk-postures for each program, which may depend on other factors such as equity concerns or type of disease for example.

## 5. Conclusions

Since decision-makers can only make informed choices when the information provided to them is comprehensive, we believe that complementing the CEAC with the CEAFC and CERAC when conducting a cost-effectiveness analysis addresses the limitations of the CEAC. The CEAFC and CERAC can easily be constructed using the results of a stochastic cost-effectiveness analysis. Real-world studies on how decision makers may use the information generated by the CEAC, CEAFC and CERAC are needed to evaluate whether risk-aversion and budget impact do influence real world decision making.

## Figures and Tables

**Table 1:** Costs and effects of two hypothetical programs.

Program	*μ_C_* ($)	*ơ_C_* ($)	*μ_E_* (QALY)	*ơ_E_* (QALY)	*p*
E	50,000	5000	10	1.3	0.4
F	90,000	15,000	13	1.1	0.8

*μ_C_* denotes mean costs, *ơ_C_* denotes standard deviation of costs, *μ_E_* denotes mean effects, *ơ_E_* denotes standard deviation of effects; normal distributions for costs and effects are assumed; correlation between costs and effects of each program is denoted by *p*; QALY denotes quality-adjusted life-years.

## Data Availability

R code for hypothetical data available on request from the authors.
